# Vasculogenesis from Human Dental Pulp Stem Cells Grown in Matrigel with Fully Defined Serum-Free Culture Media

**DOI:** 10.3390/biomedicines8110483

**Published:** 2020-11-09

**Authors:** Jon Luzuriaga, Jon Irurzun, Igor Irastorza, Fernando Unda, Gaskon Ibarretxe, Jose R. Pineda

**Affiliations:** 1 Cell Biology and Histology Department, University of the Basque Country (UPV/EHU), 48940 Leioa, Spain; jon.luzuriaga@ehu.eus (J.L.); jirurzun002@ikasle.ehu.eus (J.I.); igor.irastorza@ehu.eus (I.I.); fernando.unda@ehu.eus (F.U.); 2 Achucarro Basque Center for Neuroscience, University of the Basque Country (UPV/EHU), 48940 Leioa, Spain

**Keywords:** stem cells, DPSCs, neovasculogenesis, endothelial cells, Matrigel, vasculature

## Abstract

The generation of vasculature is one of the most important challenges in tissue engineering and regeneration. Human dental pulp stem cells (hDPSCs) are some of the most promising stem cell types to induce vasculogenesis and angiogenesis as they not only secrete vascular endothelial growth factor (VEGF) but can also differentiate in vitro into both endotheliocytes and pericytes in serum-free culture media. Moreover, hDPSCs can generate complete blood vessels containing both endothelial and mural layers in vivo, upon transplantation into the adult brain. However, many of the serum free media employed for the growth of hDPSCs contain supplements of an undisclosed composition. This generates uncertainty as to which of its precise components are necessary and which are dispensable for the vascular differentiation of hDPSCs, and also hinders the transfer of basic research findings to clinical cell therapy. In this work, we designed and tested new endothelial differentiation media with a fully defined composition using standard basal culture media supplemented with a mixture of B27, heparin and growth factors, including VEGF-A165 at different concentrations. We also optimized an in vitro Matrigel assay to characterize both the ability of hDPSCs to differentiate to vascular cells and their capacity to generate vascular tubules in 3D cultures. The description of a fully defined serum-free culture medium for the induction of vasculogenesis using human adult stem cells highlights its potential as a relevant innovation for tissue engineering applications. In conclusion, we achieved efficient vasculogenesis starting from hDPSCs using serum-free culture media with a fully defined composition, which is applicable for human cell therapy purposes.

## 1. Introduction

The establishment of an intricate vasculature network is essential to provide cellular nutrients and sustain the adequate growth and regeneration of injured tissues and organs. Blood vessels can be generated from the sprouting and expansion of pre-existing ones (angiogenesis) or by de novo differentiation of endothelial cells and the in situ formation of new vasculature (vasculogenesis). The promotion and control of both processes is paramount for tissue engineering. Innovative strategies based on stem cells hold great promise for generating functional and histo-integrative blood vessels [1]. To this end, choosing the appropriate stem cell source is important. Reduction of host immune reaction, in vitro expansion ability, ease of isolation and the capacity to successfully integrate within the grafted tissue are all factors to consider [2]. The use of autologous vascular cells extracted from human blood has been used as a very practical and handy solution to obtain high quantities of endothelial cells [3,4]; however, the low efficiency of vasculogenesis induced by the in vivo transplantation of purified endothelial cells has prompted the design of more elaborate strategies. Currently, both endothelial and perivascular cells are usually combined in the graft [5], because the successful generation of new blood vessels requires the presence of endothelial cells as well as pericytes, which constitute the mural cell layer. The interaction of both cell types is necessary to generate functional blood vessels [1]. Thus, these combinatory approaches usually employ endothelial and mesenchymal stromal cells (MSCs), where the MSCs give rise to the mural vascular component [6]. With regard to other possible sources of vasculogenic stem cells, induced pluripotent stem cells (iPSCs) are theoretically able to generate cells of any tissue [7]. However, despite their immense potential, the use of iPSCs for medical use and human therapy is currently not possible due to the inherent risks of incomplete reprogramming of the cells and de novo mutations [8]. For this reason, the use of adult autologous and non-tumorigenic stem cells still remains the best approach for vascular cell therapy.

Neural-crest derived stem cells from human dental pulp (hDPSCs) are a particularly interesting cell type for human therapy because of their multifaceted characteristics and easy accessibility [9]. These cells were initially described as one specific type of ecto-mesenchymal stromal cells, because they fulfill the morphological and marker expression criteria for their classification as such, and they can also be induced to differentiate to diverse connective tissue lineages when they were maintained in culture media containing 10–20% fetal serum [10,11]. The use of hDPSCs created substantial interest in the tissue engineering field because of their high capacity for multilineage differentiation [12,13]. However, it soon became apparent that hDPSCs were not a conventional type of MSCs, and that their capacities exceeded those of a typical MSC. Because of their neural crest origin, hDPSCs can also be induced to adopt a very large diversity of non-mesenchymal cell lineage commitments, particularly when they grow under serum-free culture conditions [14]. Some of the non-mesenchymal differentiation pathways from hDPSC include the generation of Schwann cells [15], and vascular endothelial and pericyte cells [16].

Previous works showed the ability of DPSCs to co-differentiate to both osteoblasts and endothelial cells [17,18]. However, one of the main limitations of these studies was the use of fetal serum in the cell culture medium. Fetal bovine serum (FBS) is a typical component of standard hDPSC culture media that is used to improve cell survival and expansion in vitro. The presence of animal fetal serum was also deemed necessary for the endothelial differentiation of hDPSCs [19,20]. However, the hypothetical presence of xenogenic fetal serum in a cell therapy regime would cause graft rejection [21,22,23,24] as well as uncontrollable outcomes of cellular behavior in vivo [24,25,26]. Notably, it was shown that the long-term presence of fetal serum induced the osteo/odonto-differentiation of hDPSCs [25,26]. For all the above mentioned reasons, the use of fetal serum is strongly discouraged for human cell therapies [24]. In our previous work, we described an alternative culture method using Neurocult™ media (Stem Cell Technologies), which in its Neurocult NS-A proliferation form, induced the production of endothelial and pericyte cells from human DPSCs (hDPSCs), which generated free-floating dentospheres without any need for fetal serum at any stage of the cell culture procedure [16]. This culture medium, henceforth termed Neurocult proliferation, contained a mixture of B27 without vitamin A, heparin, growth factors EGF and bFGF, and a proliferation supplement of undisclosed composition at a ratio of 9:1.

In the present work, we go a step further to characterize the vasculogenic properties of a new completely defined serum-free medium that contains the same basal components of Neurocult proliferation, but with the notable exclusion of the proliferation supplement, which is replaced with different concentrations of vascular endothelial growth factor, VEGF_165_. We assessed the in vitro vascular differentiation of hDPSCs and tubulogenesis in a Matrigel 3D assay. VEGF activity is widely known to play an important role in endothelial maturation, angiogenesis and neovascularization [27,28,29]. Human VEGF (VEGF-A) exists in several isoforms including VEGF_l2l_, VEGF_165_, VEGF_189_ or VEGF_206_ generated from alternative exon splicing of a single VEGF gene following its signal sequence cleavage [28]. VEGF_206_ is a very rare form that is only identified in the human fetal liver [30]. VEGF_189_ and VEGF_206_ are almost completely sequestered by the ECM [31]. Finally, both VEGF_121_ and VEGF_165_ are secreted and freely diffusible proteins that become VEGF_165_ (VEGF-A_165_), the predominant molecular species [30] and the most relevant biologically active isoform in physiological as well as pathological angiogenesis [32]. The rest of the components and growth factors included in the defined medium recipe (EGF, bFGF) were part of the original composition of Neurocult proliferation, which allowed for the adequate growth of hDPSCs without the use of fetal serum while maintaining the capacity of these cells for neural differentiation [16].

In the present work, we managed to generate endothelial capillary-like structures positive for endothelial cells from hDPSCs cultures in a completely defined media composition in the absence of serum. The clinical relevance is major because we have established a fully defined protocol with the total absence of fetal serum to obtain vascular cells starting from cultures of human stem cells isolated from dental pulp.

## 2. Materials and Methods

### 2.1. Cell Culture

Human third molars were obtained from healthy donors of between 19 and 45 years of age after their informed written consent. All the procedure was officially approved according to the 14/2007 Spanish Directive for Biomedical Research and the CEISH committee of UPV/EHU (Ethics committee of the University of the Basque Country) under the M10_2016_088 protocol, and abiding by the ethical principles of the Declaration of Helsinki on medical research involving human subjects. hDPSC isolation and culture was performed as previously reported [16]. Third molars were mechanically fractured to obtain dental pulp tissue. Then, a solution containing 3 mg/mL collagenase (cat#17018-029, Thermo Fisher Scientific, Waltham, MA, USA), and 4 mg/mL dispase (cat#17105-041, Thermo Fisher Scientific, Waltham, MA, USA) in Hank’s Balanced Salt Solution (HBBS) (14025092, Thermo Fisher Scientific, Waltham, MA, USA) was used to digest the pulp tissue enzymatically for 1 h at 37 °C. After centrifugation at 1200 rpm for 10 min, the pulp was mechanically dissociated by 18-G needles (304622, BD Microlance 3). DPSCs were cultured in routinely used serum-free Neurocult™ NS-A Proliferation culture medium. This medium was composed of human Neurocult NSA basal medium (cat# 05750, Stem Cell Technologies, Vancouver, BC, Canada) with a Neurocult proliferation supplement of undisclosed composition (cat# 05753, Stem Cell Technologies, Vancouver, BC, Canada) at a 9:1 ratio, supplemented with Heparin solution 2 mg/mL (cat#07980, Stem Cell Technologies, Vancouver, BC, Canada), EGF 20 ng/mL, and FGFb 10 ng/mL (Peprotech, London, UK) as previously described [33]. To minimize the risk of bacterial contaminations, penicillin 100 U/mL and streptomycin 150 mg/mL (15140-122, Gibco, Karlsruhe, Germany) were added to each culture. DPSCs were expanded in low attachment T-25 flasks and they were maintained at standard conditions in a humidified 37 °C incubator containing 5% CO_2_. Dentosphere cultures were then passaged every 7 days by enzymatic disaggregation with Accutase (cat#7920, Stem Cell Technologies, Vancouver, BC, Canada). Cell counting was performed after cell dissociation by an automatic TC20 from Bio-Rad cell counter. We cultured DPSCs cells for 1 month and a maximum of four total passages in order to avoid cell aging issues. To avoid donor variability issues, we performed all the assays with different media in parallel using cells from the same donors.

Mouse liver sinusoidal endothelial cells (*m*LSECs) were washed and cultured with RPMI 1640 medium (cat#R7388, Sigma-Aldrich; St. Louis, MO, USA) supplemented with 5% FBS (cat#SV30160.03, Hyclone, GE Healthcare LifeSciences, Logan, UT, USA) and 1% of endothelial cell growth supplement (ECGS) modifying the protocol previously described [34]. LSECs were seeded in T25 flasks previously coated with collagen I (cat#C2249 Sigma-Aldrich; St. Louis, MO, USA). Cells were passaged using trypsin-EDTA (cat#25200-072, Gibco, Karlsruhe, Germany).

### 2.2. Determination of Number and Size of hDPSC Dentospheres

To measure the number and size of hDPSC-derived spheres, these were disaggregated at passage (P) 1 or 2 using accutase (Sigma, St. Louis, MO, USA). hDPSCs were then seeded at a density of 2000 cells per well in Neurocult NSA basal medium with commercial Proliferation supplement or replacing the supplement with either high (100 ng/mL) or low (10 ng/mL) concentrations of VEGF-A_165_ (#78159.1, Stem Cell Technologies, Vancouver, BC, Canada). After 7 days in vitro (DIV), random image snapshots using 20X magnification (area of 976,023 μm^2^ per field) were taken and the average sphere number and size per condition was quantified using FIJI software (https://fiji.sc/ University of Wisconsin-Madison) [35,36].

### 2.3. Endothelial Differentiation of hDPSC

Dentospheres from P1 or P2 were disaggregated using accutase (Sigma, St. Louis, MO, USA) and hDPSCs were seeded at a density of 25,000 cells/well on laminin-coated coverslips (1:100, cat#L2020 Sigma-Aldrich; St. Louis, MO, USA) in a 24-well plate with Neurocult proliferation medium (composition previously described in Section 2.1) for 24 h to allow the cells to attach. Then, the culture medium was washed and replaced with different serum-free conditions: (i) Control Neurocult NSA basal medium with commercial Proliferation supplement as previously mentioned, (ii) Neurocult NSA basal medium without Proliferation supplement combined with a high concentration of VEGF-A_165_ (100 ng/mL; VEGFh) (#78159.1, Stem Cell Technologies, Vancouver, BC, Canada), (iii) Neurocult NSA basal medium without Proliferation supplement combined with a low concentration of VEGF-A_165_ (10 ng/mL; VEGFl), and (iv) Plain Neurocult NSA basal medium without Proliferation supplement nor any concentration of VEGF-A_165_. Cells were allowed to differentiate for 7DIV.

### 2.4. Matrigel Cultures of hDPSCs

For endothelial induction on Matrigel, coverslips on the wells of 24-well plates were covered with 500 µL of Matrigel (Ref.356234, BD Bioscience, San Jose, CA, USA) diluted 1:1 with the above-mentioned culture media conditions. Either hDPSCs or LSEC were seeded at a density of 50,000 cells/well and cultured in a humid atmosphere with 5% CO_2_ for 48–72 h. The formation of capillary-like structures was observed over time using an inverted optical Zeiss Primovert microscope and pictures were taken at different DIV using an Axiocam ERc 5s camera.

### 2.5. Tube Formation Analysis

Bright field images were acquired at different culture days, from 0 to 7 DIV. Image processing and analysis were made using FIJI software (https://fiji.sc/ University of Wisconsin-Madison) [35,36] with calibrated images. For tube formation quantification (tube number and branching points) the “Cell counter” plug-in was run to make a manual tube count, followed by branching point identification with the same program. The software automatically carried out the tube classification based on branching point quantity. We used the “Segmented line” and “polygon selection” tools to measure tube length and area, respectively.

### 2.6. Immunofluorescence (IF)

Dissociated DPSCs were seeded in laminin-treated coverslips (L2020, Sigma, St. Louis, MO, USA). After 1 week, they were fixed with 4% PFA for 10 min at room temperature. Samples were incubated for 15 min with 0.1% Triton X-100 for permeabilization, and then incubated overnight at 4 °C with primary antibodies at the following dilutions: anti-CD31 (550247, 1:300 BD Pharmingen, San Jose, CA, USA), vWF (ab6994, 1:400 Abcam, Cambridge, UK). Secondary antibodies conjugated to Alexa 488 and 568. Donkey anti-mouse, anti-rabbit in 1:200 dilution were incubated for 2 h at room temperature. Preparations were counterstained with DAPI (1:1000) and images were captured using a Leica S800 confocal microscope at X20 magnification.

Immunofluorescence in Matrigel was performed after sample fixation in 4% pre-warmed PFA for 20 min at room temperature. Next, permeabilization and primary antibody incubation steps were performed as previously described. Secondary antibodies were also incubated for 2 h at room temperature, and the samples were counterstained with DAPI (1:1000). Samples were stored at room temperature to keep the consistency of the Matrigel.

### 2.7. Flow Cytometry

The analysis was performed as previously described [16]. Briefly, a half-million hDPSCs cultured as dentospheres in Neurocult proliferation media were disaggregated. Then, they were incubated with a PBS 0.15% BSA solution containing antibodies conjugated with fluorophores. Mesenchymal stem markers CD90-FITC (#328107, 1:50 Biolegend, San Diego, CA, USA), CD105-PE 1:50 (#12-1057-41, eBioscience, Waltham, MA, USA), and CD73-APC 1:50 (#17-0739-41, eBioscience, Waltham, MA, USA), the hematopoietic marker CD45-APC (#304011, Biolegend, San Diego, CA, USA) and the endothelial marker CD31-FITC 1:50 (#400107, BioLegend, CS, USA) were analyzed. A total of 0.5 mg of each or IgG2a k Isotype control (#303103, BioLegend, CS, USA) were added for 1 h at 4 °C. After washing with PBS 0.15% BSA, cells were resuspended in 300 µL of PBS with 0.15% BSA and analyzed by flow cytometry. Flow cytometry analysis was performed by a FACS-Beckman Coulter Gallios device (Beckman Coulter Life Sciences, Indianapolis, IN, USA). The data were processed using Flowing Software 2.5 (University of Turku, Turku, Finland).

### 2.8. Statistical Analysis

Comparisons between multiple groups were made using Kruskal-Wallis followed by Dunn’s post hoc test, except for those cases showing a normal distribution, which were carried out by one-way ANOVA, followed by the Holm-Sidak post hoc test. Finally, comparisons between only two groups were made by the non-parametric Mann-Whitney U test. * *p* < 0.05, ** *p* < 0.01 and *** *p* < 0.001 were considered statistically significant. Results are shown as mean ± standard error of the mean (SEM).

## 3. Results

### 3.1. Characterization of hDPSCs Derived from Vasculogenic Dentospheres

**Figure 1:**
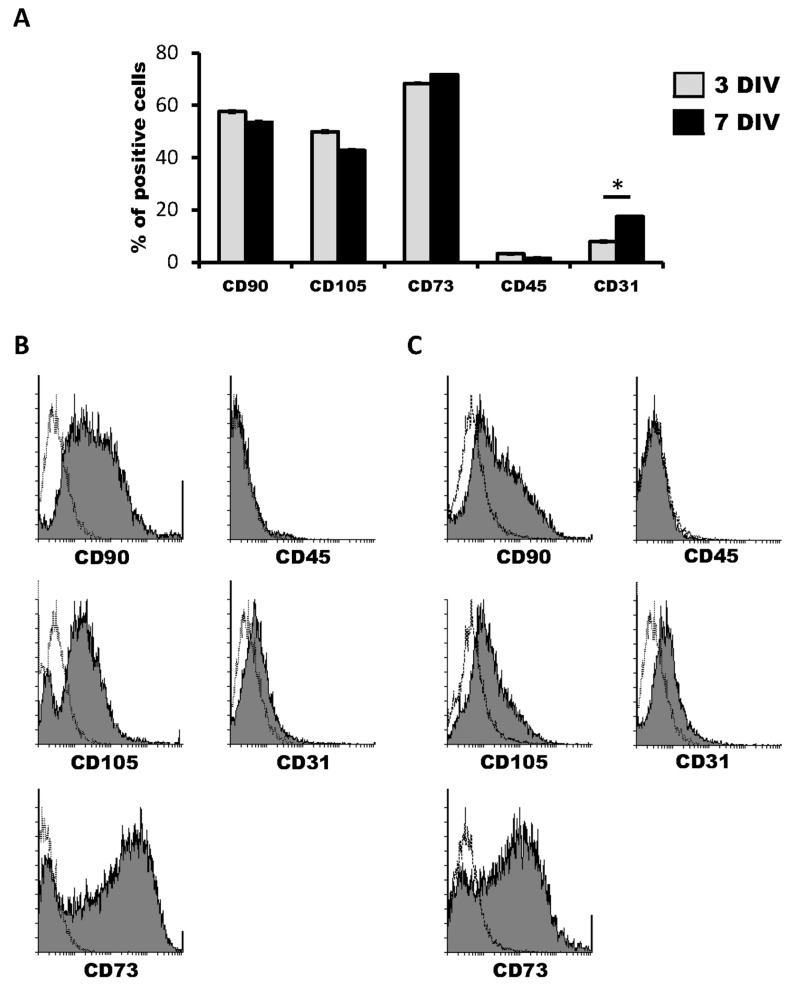
Characterization of human dental pulp stem cells (hDPSCs) cultured in Neurocult™ proliferation medium by flow cytometry. (**A**) Data quantification as representative histograms at 3 days in vitro (DIV) (gray) and 7DIV (black) for CD90, CD105 and CD73 mesenchymal stromal cell (MSC) markers, CD45 hematopoietic and CD31 endothelial markers (*n* = 3). Data are represented as the average percentage and standard error of the mean (SEM±), with respect to the total cell population. (**B**) Representative cytometry of CD90, CD105 and CD73 mesenchymal, CD45 hematopoietic and CD31 endothelial markers in hDPSCs cultured in Neurocult proliferation medium at 3DIV (gray filling), with respect to negative controls (no filling). (**C**) Representative cytometry of CD90, CD105 and CD73 mesenchymal, CD45 hematopoietic and CD31 endothelial markers in hDPSCs cultured in Neurocult proliferation medium at 7DIV (gray filling), with respect to negative controls (no filling). *: *p* < 0.05. Kruskal-Wallis with Dunn’s post hoc test.

We first characterized the cell marker expression profile of hDPSCs grown in Neurocult proliferation medium by flow cytometry. The co-expression of CD90, CD105 and CD73 markers defined a multipotent stem cell population. At three days of in vitro of cell culture, hDPSC expressed CD90, CD105 and CD73 mesenchymal stem cell markers in 57.57 ± 0.34%, 50.50 ± 0.23% and 69.09 ± 0.22% of cells within the total population, respectively. On the other hand, the positive cells’ percentage for CD45 hematopoietic marker was never higher than 2.93 ± 0.14% (Figure 1A,B). Four days later, at 7DIV, CD90, CD105 and CD73 positive cells represented 52.45 ± 0.12%, 42.39 ± 2.26% and 72.21 ± 1.97% of the hDPSC population, respectively, whereas the percentage of CD45+ cells remained very low, at 1.23 ± 0.03% (Figure 1C,D).

Regarding the endothelial marker CD31, 7.87 ± 0.04% of hDPSCs were positive for it at 3DIV. Contrary to the other tested markers, there was an increase in the proportion of CD31 positive cells at 7DIV, where 16.69 ± 0.34% of total cells were CD31+, thus more than doubling the initial percentage of positivity on the 3DIV to 7DIV interval. (Figure 1A–D). These results confirmed the presence of both mesenchymal stem cells and the previously identified CD31+ endothelial cell population in hDPSCs cultures using NeuroCult™ proliferation medium. Interestingly, it should be taken into account that it is highly likely that at least part of the cells that label neither with mesenchymal nor vascular markers may represent a population of neural-like cells [16] or the existence of some other possible yet-to be defined cell populations.

### 3.2. Sphere Generation of hDPSCs in Basal Neurocult Medium Supplemented with Either Commercial Proliferation Supplement or Different VEGF_165_ Concentrations

**Figure 2:**
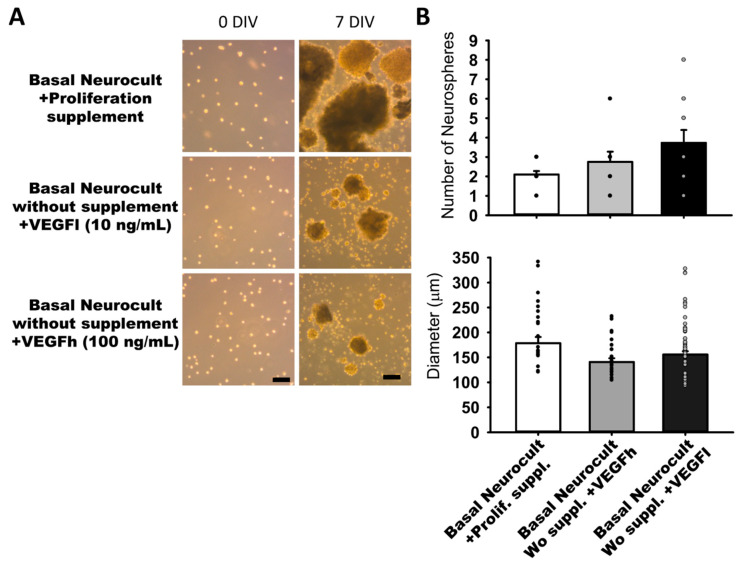
Neurocult™ proliferation supplement replacement with varying doses of vascular endothelial growth factor (VEGF) does not affect dentosphere growth. (**A**) Bright field images of dentospheres generated by hDPSCs cultured in different conditions at 0DIV and 7DIV. Scale bar = 100 µm. (**B**) Analysis of dentosphere number and diameter (µm) at 7DIV (dots indicate data from different culture wells; mean ± SEM). There were not significant differences between groups. Kruskal-Wallis with Dunn’s post hoc test.

In our search for completely defined culture media that fulfilled the requisites of vascular induction of hDPSCs while dispensing with the commercial Neurocult proliferation supplement, we addressed the potential of VEGF-A_165_ as a candidate substitute component. We performed parallel dentosphere culture assays using high (100 ng/mL, VEGFh), and low (10 ng/mL; VEGFl) concentrations of VEGF-A_165_ and control (no VEGF-A_165_) for 7DIV. In the conditions where VEGFl or VEGFh were included as a replacement for the Neurocult™ proliferation supplement, hDPSCs also grew and generated free floating dentospheres of comparable size and quantity to those formed in the full standard Neurocult™ medium (Figure 2A). There were no significant differences between the three analyzed culture conditions either in sphere number per field (2.10 ± 00.18 spheres Neurocult proliferation + supplement, 2.75 ± 00.52 spheres Neurocult basal + VEGFh and 3.73 ± 00.66 spheres Neurocult basal + VEGFl conditions) or in sphere diameter (178.42 ± 12.51 µm Neurocult proliferation + supplement, 140.75 ± 7.45 µm Neurocult basal + VEGFh and 155.83 ± 6.45 µm Neurocult basal + VEGFl conditions (Figure 2B).

### 3.3. Endothelial Differentiation of hDPSCs in Different Culture Conditions

**Figure 3:**
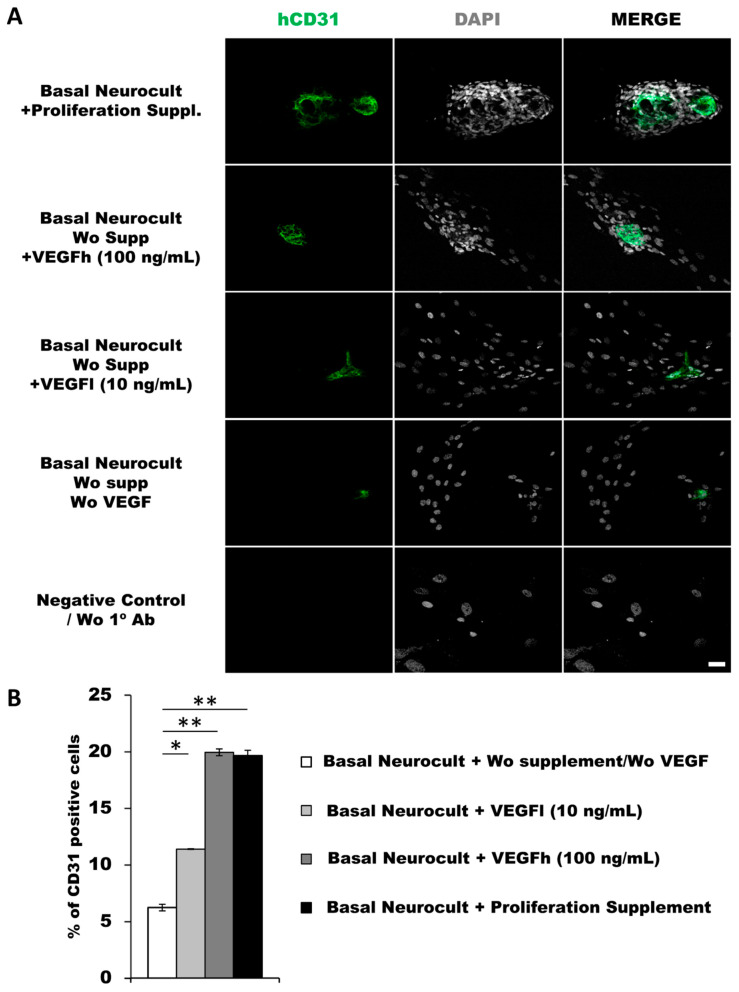
hDPSCs grown in Neurocult proliferation and basal media supplemented with VEGF_165_ show endothelial differentiation capacity under adherent conditions (**A**) Representative immunofluorescence images for CD31 endothelial marker of previously selected cell culture conditions. Gray color; DAPI. Green; CD31. Scale bar = 100 µm. (**B**) Quantification of CD31 relative positive cell percentage for each culture condition at 7DIV (*n* = 3). Means in % and standard error of the mean (SEM±) are shown. *: *p* < 0.05; **: *p* < 0.01. Kruskal-Wallis with Dunn’s post hoc test.

**Figure 4:**
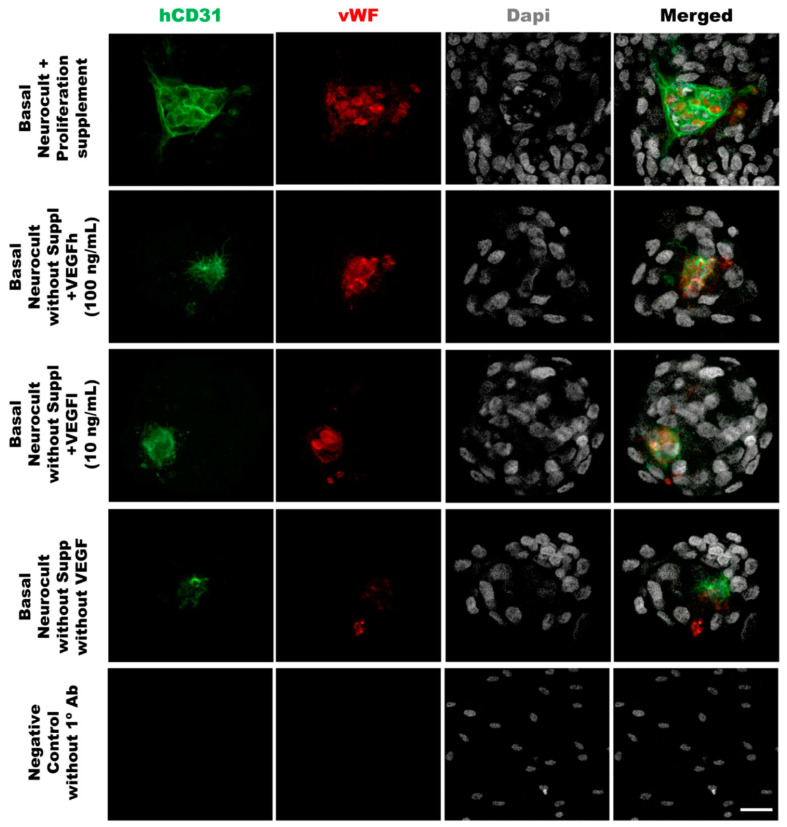
**Double immunostaining against vWF and CD31 endothelial markers of hDPSCs grown in the different culture media conditions**. Representative immunofluorescence images for CD31 and vWF endothelial markers of previously selected cell culture conditions. Gray color; DAPI. Green; CD31, Red:vWF. Scale bar= 20 µm.

As another confirmation of the results obtained by flow cytometry, we decided to assess endothelial marker expression by immunocytochemistry (ICC) on laminin-coated coverslips. hDPSCs seeded under these adherent conditions in all of the different culture media showed CD31+ endothelial cells (Figure 3A). Data for immunofluorescence showed a significantly higher percentage of positive CD31+ cells after 7DIV in hDPSCs cultures grown in either standard Neurocult proliferation (19.66 ± 0.48%) or basal Neurocult with VEGFh (19.96 ± 0.29%), compared to VEGFl, (11.39 ± 0.03%) and hDPSCs cultured in plain basal Neurocult™ with no proliferation supplement nor VEGF-A_165_. (6.23 ± 0.28%) (Figure 3B). Furthermore, a complementary staining against vWF endothelial marker, co-labeled CD31/vWF+ cells in all the studied conditions (Figure 4).

### 3.4. Tubulogenesis Ability of hDPSCs in a Matrigel 3D Culture

**Figure 5:**
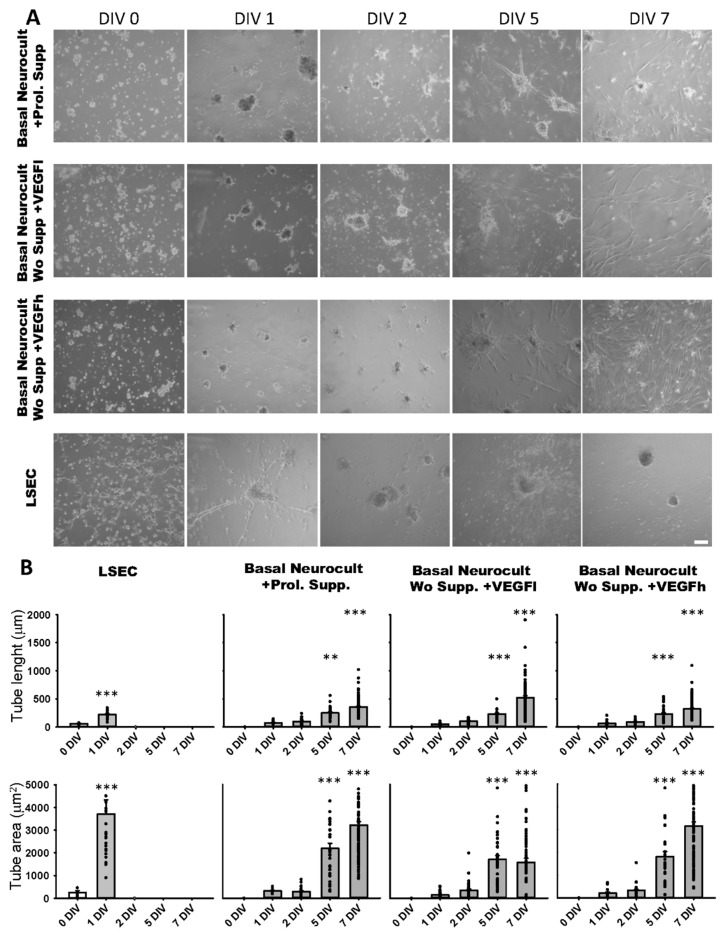
hDPSCs grown in VEGF-supplemented basal media generate 3D vascular tubules in Matrigel. (**A**) Bright field images of the tubule-like vascular network in Matrigel generated by hDPSCs cultured in different conditions from 0DIV to 7DIV. Liver sinusoidal endothelial cells (LSEC) were used as an endothelial cell positive control. Scale bar = 100 µm. (**B**) Quantification of tube length (µm) and area (µm^2^) mean and SEM ± analysis of vasculature-like structure of hDPSCs and LSECs in Matrigel from 0DIV to 7DIV (*n* = 66 for LSEC, *n* = 198 for Neurocult proliferation, *n* = 211 for VEGFh and *n* = 265 for VEGFl). **: *p* < 0.01; ***: *p* < 0.001. ANOVA Holm-Sidak post hoc test.

The formation of capilar-like tubular structures in Matrigel™ is one of the most widely used techniques to assess and quantify angiogenesis capacity in vitro. We assessed the ability of DPSC-derived endothelial cells to create 3D vascular tubules on Matrigel during 7DIV cultures (Figure 5A). We selected the timepoint of best tubule formation and stability for morphometric analysis. On dissociated hDPSC cultures, the commercial Proliferation supplement™, VEGFh and VEGFl were added to Neurocult™ NSA basal medium. We previously demonstrated that these three culture media were able to provide dentospheres containing CD31+ cell populations. Endothelial cells derived from hDPSCs cultured in Neurocult™ proliferation supplement gave rise to capilar-like structures as early as after 24 h of culture: tube length 76.76 ± 08.87 µm, 96.91 ± 7.71 µm, 250.25 ± 17.13 µm and 358.16 ± 14.26 µm for 1, 2, 5 and 7 DIV, respectively, (*p* < 0.05 and *p* < 0.001 for 5DIV and 7DIV respectively, one-way ANOVA). The tube area was 313.33 ± 36.42 µm^2^, 275.86 ± 25.44 µm^2^, 2251.21 ± 226.58 µm^2^ and 3210.14 ± 151.80 µm^2^ for 1, 2, 5 and 7 DIV, respectively (*p* < 0.001 for 5 and 7DIV, one-way ANOVA). For hDPSCs grown with Neurocult with VEGFh, the values for the tube length were: 63.12 ± 9.84 µm, 90.93 ± 5.16 µm, 230.23 ± 23.26 µm and 322.82 ± 14.84 µm for 1, 2, 5 and 7 DIV, respectively (*p* < 0.001 for 5 and 7DIV, one-way ANOVA; Figure 5B). The tube area was 208.65 ± 47.37 µm^2^, 367.77 ± 58.10 µm^2^, 2079.81 ± 272.01 µm^2^ and 3180.48 ± 386.03 µm^2^ for 1, 2, 5 and 7 DIV, respectively (*p* < 0.001 for 5 and 7DIV, one-way ANOVA). Interestingly, VEGFl also showed a similar tubulogenesis rate over time as the rest of the conditions: tube length 50.29 ± 03.05 µm, 103.19 ± 06.56 µm, 232.25 ± 17.21 µm and 521.55 ± 35.91 µm for 1, 2, 5 and 7 DIV, respectively (*p* < 0.001 for 5 and 7DIV, one-way ANOVA). The tube area was 138.42 ± 21.34 µm^2^, 309.15 ± 36.90 µm^2^, 1792.81 ± 205.77 µm^2^ and 1788.65 ± 193.69 µm^2^ for 1, 2, 5 and 7 DIV, respectively (*p* < 0.001 for 5 and 7DIV, one-way ANOVA, Figure 5B). Liver sinusoidal endothelial cells (LSECs) were used as an endothelial cell positive control and we performed parallel assays using LSECs (Figure 5A). LSECs generated tubules much faster than hDPSCs in the short term, attaining maximal tubulogenesis at 1DIV with an average tube length of 58.27 ± 7.82 µm and 222.56 ± 13.80 µm for 0 and 1DIV, respectively (*p* < 0.001 one-way ANOVA) and tube area of 256.05 ± 72.00 µm and 3704.90 ± 618.60 µm for 0 and 1DIV, respectively (*p* < 0.001 one-way ANOVA). However, the fast growth of LSEC capilar-like structures was not sustained from 2DIV onwards because cells rapidly lost their viability. In conclusion, the quantified tubulogenesis parameters of hDPSCs achieved at 5 and 7DIV were comparable to those of 1DIV for LSECs.

**Figure 6:**
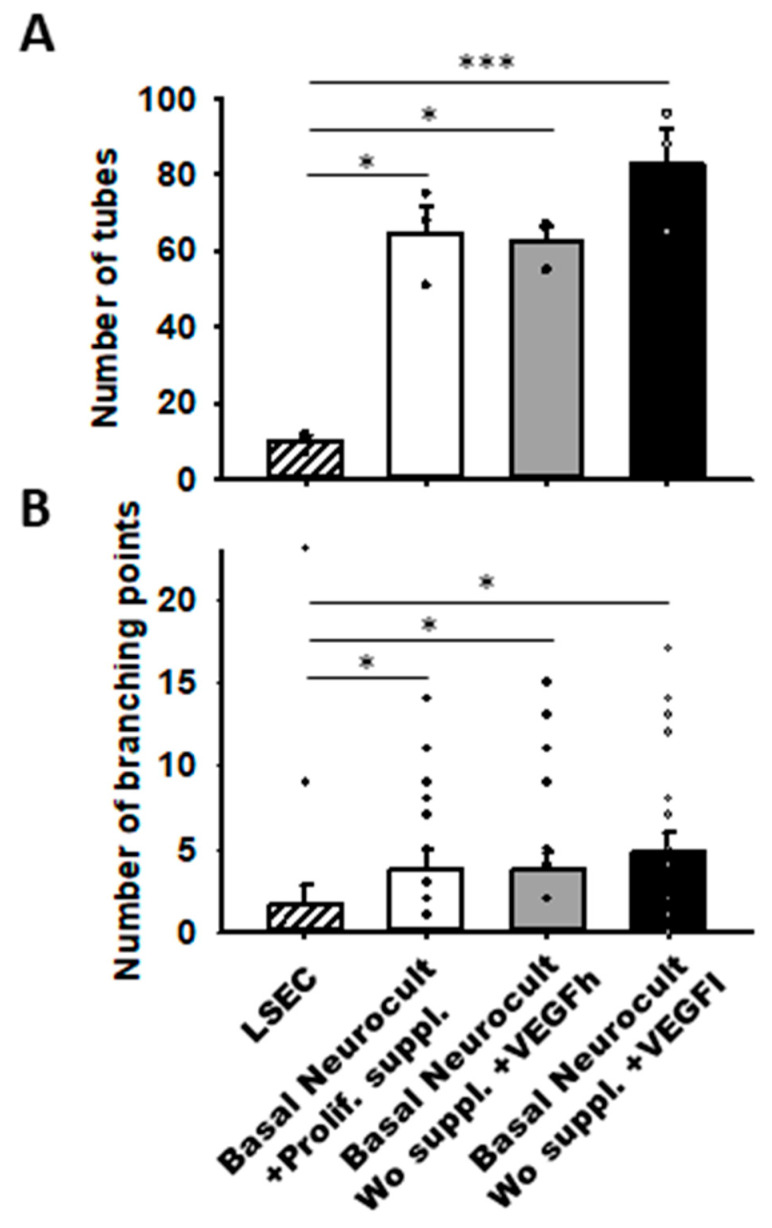
VEGFh/l-supplemented basal media generate vascular structures in similar proportion to commercial supplemented Neurocult proliferation medium. (**A**) Quantification of the number of tubes/mm^2^ plotted as mean ± SEM (*n* = 4 aleatory regions) at 5DIV for hDPSCs and 1DIV for LSECs. (**B**) Quantification of branching points expressed as mean ± SEM at 5DIV for hDPSCs and 1DIV for LSEC (*n* = 21 aleatory regions). *: *p* < 0.05; ***: *p* < 0.001. Kruskal-Wallis with Dunn’s post hoc test.

Remarkably, we observed that hDPSCs grown either with proliferation supplement or with both h/L doses of VEGF at 5DIV created more capilar-like structures than those observed for LSECs seeded at a comparable density: 64.66 ± 7.12 tubes/mm^2^ in DPSCs cultured with Neurocult proliferation supplement, 62.66 ± 3.84 tubes/mm^2^ for Neurocult with VEGFh, and 83.00 ± 9.29 tubes/mm^2^ for Neurocult with VEGFl compared to barely 10.00 ± 1.52 tubes/mm^2^ observed for LSECs (*p* < 0.05 and *p* < 0.001 Kruskal-Wallis, Figure 6A). Next, we measured the interconnections or branching points of the tubes and we found an average of 3.80 ± 1.09 branches/mm^2^ for Neurocult with proliferation supplement, 3.70 ± 1.14 branches/mm^2^ for Neurocult with VEGFh, and 4.75 ± 1.22 branches/mm^2^ for Neurocult with VEGFl compared to 1.60 ± 1.21 branches/mm^2^ for LSEC (*p* < 0.05 one-way ANOVA, Figure 6B).

### 3.5. Endothelial Characterization of hDPSC-Generated 3D Vasculature

**Figure 7:**
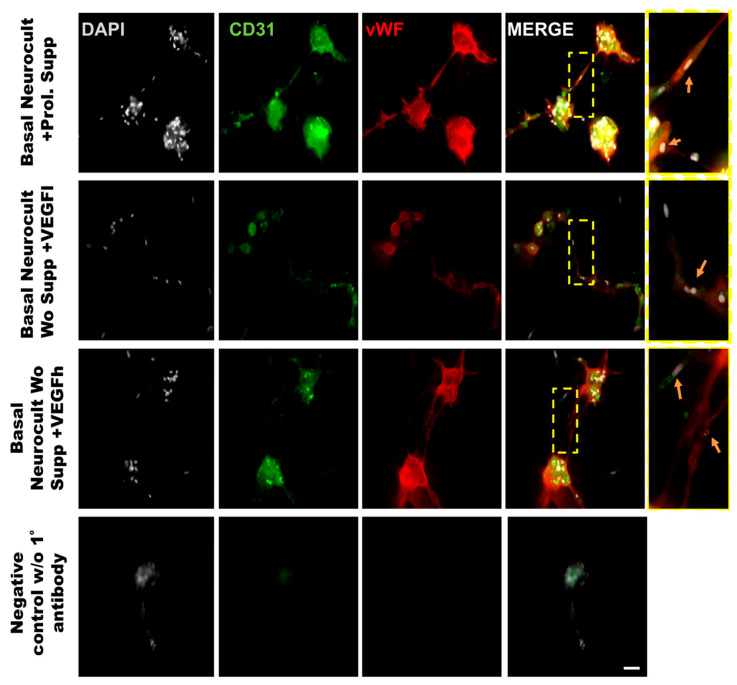
Representative immunofluorescence images of the neovasculature network generated by hDPSCs in different growth conditions in Matrigel. Scale bar = 100 µm. Gray color; DAPI. Green color; CD31 endothelial marker. Red color; vWF endothelial marker. Yellow box roi; digital magnification. Scale bar = 30 µm. Yellow arrow; hDPSCs expressing CD31 and vWF.

In order to demonstrate that the tubes formed from hDPSCs in vitro using Matrigel were capilary-like structures, we assessed the cellular phenotype by co-immunostaining against the endothelial CD31 and vWF (von-Wildebrand Factor) markers. The immunostaining of the tubes was made at 5DIV, one of the timepoints where the number of tubes and branching were quantified. The results demonstrated the ability of hDPSCs to create tubes between clusters of hDPSCs, mimicking the behavior observed in endothelial cell lineages such as LSECs (Figure 7). Our results confirmed that hDPSCs grown in Neurocult basal medium either with commercial proliferation supplement or alternatively, in the presence of VEGF_165_ were able to produce CD31+ and vWF+ capilary-like structures with the characteristics of genuine vasculature (Figure 7, yellow arrows).

## 4. Discussion

**Figure 8:**
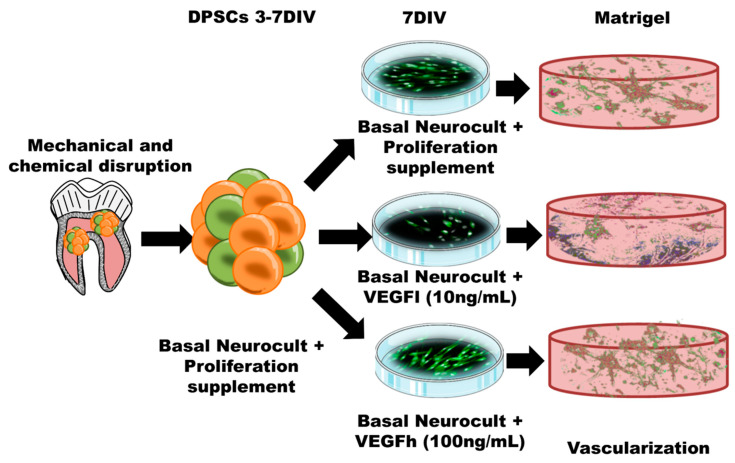
Schematic illustration of the protocol followed for hDPSC expansion, culture and differentiation both in 2D culture plates and in 3D Matrigel. Both the 2D monolayer growth mode in standard culture plates and the 3D vascular-like network growth in Matrigel are represented.

In our previous work, we found that hDPSCs grown in Neurocult proliferation medium were able to generate cells with endothelial phenotype, with the ability to graft and generate neovasculature into the brain of athymic nude mice. However we did not demonstrate their physiological functionality (e.g., presence of blood) [16]. Given the unexpected nature of this result (as no specific endothelial culture media were employed) and the unknown components present in the Neurocult Proliferation Supplement™, we further sought to reproduce the endothelial induction effect on hDPSCs, but this time using a completely defined medium. Neurocult NSA proliferation is a serum-free culture medium enriched with a proliferation supplement with non-disclosed compounds, which allows the survival and growth of hDPSCs as free-floating dentospheres [16]. In the present work, we describe the possibility of keeping similar levels of hDPSC viability and endothelial induction while avoiding the use of fetal serum and by replacing the proliferation supplement with VEGF-A_165_ at different concentrations. We demonstrate, as a proof of concept that this simple modification is sufficient to maintain the viability of dentospheres from hDPSCs and their potential to generate endothelial cells and vascular tubules in 3D culture (Figure 8). However, it should be taken into account that high levels of VEGF may be responsible for the development of vascular hyper-permeability and accelerated tumor development [37,38]. For these reasons, we focused this research on a proof of concept of an in vitro vasculogenesis model and relegated cell grafting as a more mature therapy to fine-tune beyond the scope of this manuscript.

One of the best tools to test angiogenesis and vasculogenesis in vitro are Matrigel 3D cultures. Matrigel is an extract of the Engelbreth-Holm-Swarn sarcoma that contains basement membrane components able to induce the formation of tube-like structures by different endothelial cells [39,40,41]. It has been described as the highly reproducible in vitro gold-standard for angiogenesis and vasculogenesis assays based on the formation of tube-like structures [42,43,44,45]. This assay has been widely used to screen for vasoactive compounds. Previous data have demonstrated that endothelial cells from human umbilical cords as well as from other sources differentiate and form capillary-like structures on Matrigel in the presence of 5–20% of fetal serum and 1 mg/mL of ECGS [46]. Schechner and colleagues were among the first to use HUVECs resuspended in a collagen/fibronectin gel and incubated for 20 h in vitro to obtain tubular structures before subcutaneous implantation into immunodeficient SCID/beige mice. Their analysis revealed functional vessels that had the characteristics of capillaries, venules and arterioles [47]. Since then, numerous works have used Matrigel as an in vitro assay to characterize angiogenesis [43], even in tumor cells [48]. Interestingly, Matrigel has been reported to be osteogenic for mesenchymal stem cells [49]. It is noteworthy that the Matrigel test includes the presence of fetal serum in the medium [40,46,50], which has also been demonstrated to induce the osteoblastic differentiation of DPSCs [25,26]. For these reasons, we can speculate that the original protocol would favor osteogenesis due to the presence of serum. Indeed, when bone lineage cells were cultured in Matrigel they ceased proliferation and formed canaliculi [51].

In this work, we wanted to assess the potential of hDPSCs to generate new vasculature in Neurocult basal medium completely devoid of serum and/or ECGS, and we replaced the undisclosed proliferation supplement with VEGF. Our findings demonstrate that the replacement of proliferation supplement with VEGF-A_165_ is fully compatible with both cell survival and endothelial differentiation of hDPSCs when cultured in NeuroCult™ NS-A Basal Medium. VEGF has been reported to be neuroprotective at a dose of 50 ng/mL for neuronal-like HN33 cell lines [52]. Thus, we aimed to determine the effects of VEGF in sub-neuroprotective conditions (10 ng/mL, VEGFl) and over-neuroprotective conditions (100 ng/mL, VEGFh). Our results shows that in the presence of both doses of VEGF, hDPSCs are able to form dentospheres in a similar way that we showed previously using NeuroCult™ NS-A proliferation medium [16]. Moreover, VEGF_165_ is the most relevant biologically active isoform in physiological as well as pathological angiogenesis [32], and a highly specific mitogen for endothelial cells that has been reported as a key regulator for angiogenesis [53]. We had previously demonstrated that hDPSCs express the VEGFR receptor [16]. VEGF-A_165_ signaling in HUVECs is characterized by the activation of multiple signaling effectors including PLCγ1 and the regulation of calcium ion flux to regulate cell migration [54]. Other reports point to VEGF as the main triggering signal for endothelial differentiation of vascular progenitors [55] and stem cells [56,57]. In our work, we found that VEGF-A_165_ was able to sustain the growth and survival of endotheliocytes derived from hDPSCs cultured in basal conditions without serum, EGCS or Neurocult proliferation supplement (Figure 3A,B).

To corroborate the vascular phenotype of the tube-like structures generated by hDPSCs in Matrigel, CD31 and vWF co-immunostaining was performed directly over the Matrigel samples. CD31 is a marker of endothelial cells expressed during angiogenesis [58] and vWF is a glycoprotein largely produced and secreted by endothelial cells [59], which is important for the maintenance of hemostasis and promoting the adhesion of platelets to the sites of vascular injury [60]. Other works have used vWF staining as a unequivocal marker of endothelia in collagen assays or diaminobenzidine immunostaining [43]. Even though the generation of vascular-like networks from hDPSCs in Matrigel was already described [61], to date no study using direct in situ immunostaining for endothelial markers on these tubular-like structures in Matrigel had ever been performed. This constitutes, by itself, a significant innovation. To our knowledge, we are the first group describing double in situ IF labeling on Matrigel for two different endothelial markers in a 3D vasculogenesis assay.

Vascularization is one of the most challenging issues in obtaining the effective integration of a graft into the host tissue. Indeed, the efficiency of tissue engraftment can be compromised depending on the host tissue vascularization capacity [62]. An extensive study is currently devoted to the development of vascularized engraftments in vitro, which would help to support the establishment of a dense vascular network in the target tissue to regenerate [1]. In this regard, the use of hDPSCs under our current protocol could improve the capacity to promote tissue neovascularization for future cellular therapies. In agreement, the benefits of pre-vascularized tissue transplantation have also been described in different tissue engineering models. The use of pre-vascularized engraftments involving MSCs, iPSCs and other progenitor cells for muscle [63], skin [64] and liver [65] regeneration has led in all cases, to a substantially improved cell graft integration with respect to non-vascularized grafts.

## 5. Conclusions

Our results demonstrate that VEGF-A_165_ is a good substitute for the proliferation supplement contained in commercial Neurocult™ media, specifically with regard to the induction of vasculogenesis from hDPSCs. Our fully defined culture media supported hDPSC survival, growth, endothelial differentiation, and vascular tubule generation to comparable levels as Neurocult proliferation, without any need for EGCS and/or fetal serum. Furthermore, the validation of endothelial markers such as CD31 and vWF by double immunofluorescence staining directly on 3D Matrigel represents an advance in the fine characterization of cellular phenotype in 3D vascularization assays. Altogether, our results pave the way to improving and validating future vasculogenesis and angiogenesis research for next-generation tissue engineering and cell therapies.

## 6. Patents

An international patent filing (WO/2020/007878- PCT/EP2019/067769) resulted from the work reported in this manuscript.
